# Pelvic Floor Dysfunction After Hysterectomy: Moving the Investigation Forward

**DOI:** 10.7759/cureus.15661

**Published:** 2021-06-15

**Authors:** Valerie Chen, Laura Shackelford, Marta Spain

**Affiliations:** 1 Department of Obstetrics and Gynecology, Carle Illinois College of Medicine, Champaign, USA; 2 Department of Anatomy, Carle Illinois College of Medicine, Champaign, USA

**Keywords:** pelvic floor dysfunction, hysterectomy, pelvic organ prolapse, stress urinary incontinence, autonomic innervation, inferior hypogastric plexus

## Abstract

The role of hysterectomy in the development of pelvic floor dysfunction (PFD) remains widely disputed. The controversy is fueled by two key factors. The first is conflicting association studies that make it difficult to establish whether a link truly exists. Although many retrospective studies report a correlation between hysterectomy and increased risk of stress urinary incontinence (SUI) or pelvic organ prolapse (POP), prospective studies often fail to replicate these results, leading some to conclude that no association exists. However, most prospective studies do not follow up for a sufficient length of time to account for the long latency of PFD and cannot unilaterally prove the absence of an association. The second source of controversy is the absence of a plausible mechanism to explain how hysterectomy could predispose patients to PFD. In this paper, we investigate autonomic innervation and smooth muscle in the three layers of pelvic floor support and propose a mechanism through which autonomic damage from hysterectomy could predispose patients to PFD. We then identify key research areas needed to evaluate this theory. This report aims to inspire a discussion on how to further the collective understanding of the relationship between hysterectomy and PFD. Clarifying the nature of this connection could have enormous consequences in redefining the risks and benefits of hysterectomy.

## Introduction and background

Clinicians have long suspected a link between hysterectomy and pelvic floor dysfunction (PFD), but even after decades of research, this remains remarkably controversial [1-4]. Hysterectomy is one of the most common procedures in the US, yet its long-term outcomes are poorly understood and there is no consensus on whether PFD such as stress urinary incontinence (SUI) or pelvic organ prolapse (POP) should be considered among its risks. One reason for the controversy is because literature is filled with conflicting association studies that make it difficult to establish whether a link truly exists [1-4]. Another reason is that many well-known etiologies for PFD, such as childbirth-induced somatic nerve injury or muscle trauma, simply do not occur in hysterectomy [5,6]. In the absence of a plausible mechanism, it is impossible to say whether hysterectomy causes the type of damage that could contribute to PFD. Nonetheless, this topic deserves further investigation because establishing such a link would have enormous consequences in redefining the risks and benefits of one of the most common surgeries in the US.

PFD encompasses a range of problems that can cause urinary and anorectal defects in addition to those specific to the reproductive system. Here we limit our focus to SUI and POP since these are two of the most common manifestations of PFD. Doing so allows this paper to maintain a manageable scope and to focus on the mechanism by which hysterectomy may predispose patients to these consequences. Although the etiology of PFD remains incompletely understood, it is often considered a culmination of many insults over a lifetime and associated with risk factors like age, childbirth, and congenital collagen defects [7]. Nerve injury has been proposed as a contributor, but it is often dismissed in the context of simple hysterectomy because pelvic somatic nerves are considered too lateral for accidental injury and patients lack classic symptoms of autonomic nerve damage. The traditional belief is that pelvic autonomic nerve injury presents with bladder and bowel dysfunction like urinary urge or fecal incontinence. Recent literature, however, demonstrates that smooth muscle is also present in the pelvic floor’s musculofascial support [8-10], creating the possibility that autonomic damage may present in more diverse ways than formerly believed. Furthermore, autonomic nerves are more likely to be injured than their somatic counterparts because they run medially into and around the cervix through the surgical planes of hysterectomy. Together, this makes autonomic nerve and smooth muscle damage a promising focus for exploring how hysterectomy may contribute to the pathogenesis of PFD.

This review was performed using literature from Pubmed and Google Scholar. We first discuss the contributions and pitfalls of existing association studies. We then introduce a mechanism by which autonomic damage sustained in hysterectomy could exacerbate the development of PFD through smooth muscle degradation in the pelvic floor. We discuss the role of autonomic innervation or smooth muscle in each of the three layers of pelvic floor support and investigate the steps of hysterectomy that may result in autonomic nerve damage. Lastly, we identify future research directions that are crucial to evaluating this theory. In doing so, we hope to inspire a discussion on how to further the collective understanding of hysterectomy’s relationship to PFD.

## Review

Association studies

Over the years, many retrospective studies have demonstrated a correlation between hysterectomy and SUI or POP. One systemic review found an increased odds ratio of 60% for urinary incontinence in hysterectomy patients over 60 at the time of assessment [1]. Similarly, a multi-center longitudinal study showed a hazard ratio of 1.7 (95% CI, 1.6 to 1.7) for POP surgery in hysterectomy patients compared to non-hysterectomy controls [3]. The source of confusion is that prospective studies often fail to replicate these results, leading some to conclude that no association exists [2]. This is a premature conclusion because of a simple reason: follow-up is too short. Retrospective studies, though limited by imperfect controls, have nonetheless shown that the risk for PFD remains elevated for years after hysterectomy. This is consistent with our understanding of PFD onset following risk factors like vaginal childbirth, where symptoms routinely do not appear for years after the insult. Indeed, studies that investigate PFD after vaginal childbirth routinely examine patients five to 10 years after delivery to account for this delay [11]. In contrast, prospective studies on post-hysterectomy PFD rarely follow up more than two years post-op [2], so it is likely that they fail to capture the onset of PFD in a significant portion of participants. Given the known limitations, results of prospective studies should be scrutinized just as diligently as retrospective studies and cannot unilaterally prove that no association exists.

Pelvic floor autonomics and smooth muscle

In 1992, DeLancey described three levels of pelvic floor support in what has become a widespread paradigm for pelvic floor disorders [12]. The first level is made of the cardinal and uterosacral ligaments (USL), the second includes the pubocervical and rectovaginal fasciae, and the third consists of the levator ani, perineal body, and perineum. In this section, we discuss how autonomic nerves and/or smooth muscle affect each of these three levels and explain how smooth muscle injury may lead to breakdown in pelvic floor support.

The first level includes the cardinal ligament (CL) and USL, which contain vessels, nerves, connective tissue, adipose, and lymphatics and provide mechanical support to the cervix and upper vagina [13]. Autonomic fibers from the utero-vaginal plexus travel within and around these ligaments en route to organs and other smooth muscle of the pelvis. The CL and USL are susceptible to mechanical weakening, denervation of autonomic and sensory fibers, and ischemic injury as a result of hysterectomy [14].

The second level of pelvic support includes the pubocervical and rectovaginal fasciae, which are subdivisions of the endopelvic fascia responsible for supporting the bladder, upper two-thirds of the vagina, and rectum [15]. The rectovaginal fascia is a sheath of smooth muscle and collagen that connects the anterior anorectal wall to the vagina and helps prevent posterior vaginal wall prolapse. Similarly, the pubocervical fascia is a smooth muscle-containing structure that helps maintain structural integrity in the anterior vaginal wall. Failure of this fascial plane has been linked to posterior POP [16]. These tissues play an important role in Petros and Ulmsted’s Integral Theory of female urinary incontinence, which blames laxity in the vaginal wall or supporting structures for the development of urinary incontinence [17]. Because the second level fasciae are smooth muscle-containing tissues that maintain tension in the anterior and posterior vaginal wall, events that cause smooth muscle breakdown such as autonomic denervation can lead to vaginal laxity and subsequent prolapse or incontinence.

The third and final level of pelvic floor support includes the levator ani, perineal body, and perineum. The levator ani is traditionally thought to be made entirely of striated muscle, but studies have found smooth muscle in its subdivision, the pubococcygeus [8]. In the Integral Theory, the pubococcygeus plays a key role in maintaining vaginal tension via tonic contraction [17]. The presence of smooth muscle suggests that it may be partially under autonomic control rather than entirely somatic control as formerly believed; therefore, autonomic damage may diminish its ability to maintain vaginal tension and pelvic floor integrity.

Pelvic floor support is often described in terms of striated muscle and non-innervated connective tissue without consideration of smooth muscle. However, as we describe above, autonomic nerves and/or smooth muscle contribute to each of the three pelvic support levels. Therefore, damage to pelvic autonomic nerves may have a greater impact on pelvic floor support than formerly believed.

Pelvic nerve anatomy

Innervation to the female pelvis plays a central role in our discussion. Typically, pelvic floor innervation is thought to be mainly somatic due to its contribution to the striated muscle of the levator ani and coccygeal muscle. Interestingly, there is still considerable controversy about whether this comes from the pudendal nerve or from a distinct nerve originating from sacral nerve roots S3-S5 [18]. The pudendal nerve passes behind the sacrospinous ligament and exits the pelvic cavity through the greater sciatic foramen before crossing medial to the ischial spine and entering the pudendal canal. The so-called nerve to the levator ani, in contrast, crosses the superior surface of the coccygeal muscle after exiting the sacral foramina, then moves along the superior surface of the iliococcygeal muscle, dividing into branches that innervate the levator ani and coccygeal muscles.

Given the presence of both smooth and striated muscle in the pelvic floor, the nerve contributions are likely also a mix of autonomic and somatic. Sympathetic innervation to the pelvis comes from the superior hypogastric plexus (SHP), which lies anterior to the aortic bifurcation at the L5 level and runs inferolaterally where it is renamed the right and left hypogastric nerves [19]. These merge with the pelvic splanchnic nerves, which carry parasympathetics from spinal levels S2 to S4 to form the right and left inferior hypogastric plexuses (IHP).

The IHP then divides into the rectal, uterovaginal, and vesical plexuses that innervate smooth muscle of target organs in the pelvis. The rectal plexus branches posteriorly and enters the rectal wall alongside the middle rectal artery. The vesical plexus comes from the lateral division and follows branches of the vesical artery, providing innervation to the distal ureter, urinary fundus, and internal urethral sphincter. Lastly, the medial division of the IHP forms the uterovaginal plexus, which provides autonomic innervation to the reproductive organs. The precise innervation of smooth muscle in the endopelvic fascia and levator ani is not known, but it likely comes from the uterovaginal plexus given the close proximity to the cervix and uterus.

Autonomic nerve damage in hysterectomy

Autonomic fibers of the uterovaginal plexus are closely intertwined with the cervix and surrounding tissues, putting them at risk of transection during hysterectomy. The idea that hysterectomy may cause nerve damage, though seldom discussed, is not a novel concept. Lakeman et al. described four hysterectomy steps that may result in nerve injury [6]. Likewise, Butler-Manuel et al. demonstrated parasympathetic and sympathetic nerves within the USL and CL, which are frequently divided in hysterectomy [20]. Some have argued that nerve injury from hysterectomy is insignificant because only nerves to the removed cervix and uterus are affected [2]. This fails to consider, however, that autonomic nerves to the reproductive organs may also contribute to smooth muscle of the endopelvic fascia and levator ani, which still have functional roles in maintaining pelvic floor integrity after hysterectomy.

Autonomic nerve damage is not typically associated with hysterectomy because it rarely causes the dramatic symptoms of urge incontinence or sphincter insufficiency that are common after radical hysterectomy (RH). In RH, autonomic damage is primarily attributed to partial denervation of the autonomic nerve supply during extensive parametrium and vaginal cuff dissection [21], neither of which is routinely performed in non-RH. However, the absence of acute autonomic dysfunction after non-RH does not necessarily mean that no autonomic nerve damage occurs and can be explained in two ways: first, the nerve fibers at risk in hysterectomy tend to be smaller and fewer in number than those involved in RH [20], resulting in a milder potential injury. Second, because non-RH involves dissection and ligament division more medially in the pelvis compared to RH, there is less risk of injuring the vesical plexus and therefore lower likelihood of urge incontinence. The absence of autonomic dysfunction after non-RH may simply indicate a different severity and location of damage that produces less conspicuous signs of autonomic injury compared to RH.

There are three locations where innervation to pelvic floor smooth muscle may be injured during hysterectomy: the USL, CL, and pericervical endopelvic fascia. The medial trunk of the IHP forms the uterovaginal plexus, which travels through and around the USL before innervating the uterus, cervix, upper vagina, and fallopian tubes [19]. These branches are at risk during USL ligation due to transection of nerves both within the USL and those in its close vicinity. Although nerve density within the USL is highest in the lateral part of the ligament, intraoperative biopsies show that medial ligation, as performed in non-RH, can also cause nerve fiber transection [20].

Autonomic damage can also occur during division of the CL. Histological studies reveal branches of the IHP in the posterior border of the CL surgical margin in RH [22]. Though the nerve density differs at the point of ligation in RH compared to simple hysterectomy, autonomic nerves run through the entire length of the CL so transection at any point can cause some degree of nerve damage [20].

A third source of possible autonomic damage is during the division of the pericervical endopelvic fascia in accordance with extrafascial hysterectomy technique. This method, which has become more popular in recent years due to its relative ease and lower blood loss, involves removal of the pericervical fascia together with the cervix. Kaya et al. proposed that this step may damage paravaginal nerve bundles, resulting in denervation injury [23]. In the extrafascial technique, the endopelvic fascia is excised close to the cervix where the paravaginal nerve bundles in question are likely ranches of the uterovaginal plexus. The extrafascial technique may result in two-fold damage to the pelvic floor: acute injury to the endopelvic fascia, which provides structural support to the pelvic floor, as well as denervation injury to smooth muscle whose nerve supply gets transected.

Future research topics

We have so far presented a mechanism by which simple hysterectomy may contribute to the pathogenesis of PFD through injury of autonomic nerves in the pelvis. This is supported by literature findings of smooth muscle in pelvic floor support structures and anatomical evidence for possible autonomic nerve injury in hysterectomy [6,20]. This theory sets the foundation for future research to seek an understanding of hysterectomy’s contribution to the pathogenesis of PFD.

The first research need is for prospective cohort studies with a follow-up length of five to ten years to study differences in PFD outcome between hysterectomy patients and controls. An essential benefit of prospective studies over retrospective studies is their ability to control for key confounders such as preexisting SUI or POP. However, as previously discussed, currently available prospective studies are limited by insufficient follow-up periods that cannot confidently capture the incidence of PFD in participants.

The second knowledge gap is the shortage of clinical research that investigates the association between autonomic damage and PFD. Existing research on innervation in PFD heavily focuses on the pudendal nerve, with comparably few studies on autonomic innervation. Some studies note that vaginal wall samples from POP patients exhibit greater smooth muscle atrophy compared to healthy controls [10], but more research is needed to establish a correlation. This is particularly challenging because there are few metrics to assess pelvic autonomic function in women, particularly when the injury is not severe enough to cause overt symptoms.

A third knowledge gap pertains to the comparative role of smooth versus striated muscle in maintaining pelvic floor tension. Pelvic support structures such as the levator ani are traditionally thought to be controlled by only somatic innervation [19]. Though there is evidence of smooth muscle contribution to pelvic floor support [8], its clinical significance is unknown. Animal studies may help shed light on this subject. Rats have been used as models to study the relationship between somatic nerve injury and SUI due to parallels in rat and human pudendal innervation [24]. Similarly, rats have a complex hypogastric plexus that may be used to model pelvic floor autonomic innervation in humans [25]. This makes it possible to compare differences in pelvic floor integrity between rats who suffer somatic versus autonomic nerve injury. Clarifying the respective roles of smooth and striated muscle in pelvic floor integrity may help guide new therapies and prevention efforts.

The fourth and final knowledge gap is whether the intrafascial hysterectomy technique can reduce PFD-related morbidity. In the past, the intrafascial technique was suggested as a way to minimize nerve damage and PFD risk, but evidence remains scarce. Few clinical studies investigate this topic, and the ones that do follow up for too short a time to capture PFD onset in all participants [23]. Like with prospective association studies, a five to ten-year follow-up is needed to accommodate the long latency of PFD following pelvic floor insult. This is a worthwhile pursuit because there is a strong theoretical basis for the advantage of the intrafascial technique when it comes to nerve and fascial preservation, but its clinical significance remains inadequately explored. The intrafascial approach has become less popular in recent years due to high blood loss and difficult technique, but its protection of the endopelvic fascia makes it a promising candidate for reducing post-hysterectomy PFD.

## Conclusions

Despite decades of research, the relationship between hysterectomy and PFD remains ambiguous due to conflicting results from association studies and the absence of a plausible mechanism for how hysterectomy might contribute to PFD. In this paper, we propose the theory that autonomic nerve damage sustained during hysterectomy may cause progressive weakening of smooth muscle in the pelvic floor that promotes PFD. The development of PFD is a complex, multifactorial process, and it is doubtful that any single pathway or mechanism can fully explain or predict onset. However, hysterectomy-induced autonomic nerve injury may be a contributing factor that, if proven, will force us to reconsider the long-term risks of hysterectomy. One of the key barriers to understanding the relationship between hysterectomy and PFD is the absence of a mechanism, particularly because hysterectomy is unrelated to many of the well-known risk factors for PFD like age or muscle injury. In the past, researchers have discussed the possibility of nerve damage in hysterectomy, but to the best of our knowledge, this is the first time hysterectomy-induced autonomic nerve injury has been proposed as a contributor to the breakdown of pelvic floor support. Undoubtedly, additional steps are needed to evaluate this theory and create active solutions. The first step is to close the research gap. Studies should include prospective studies with increased follow-up periods, evaluation of autonomic damage in PFD, further investigation into the respective roles of smooth and striated muscle in pelvic floor support, and clinical studies to assess whether intrafascial and extrafascial hysterectomy cause different PFD outcomes. The second step is to be cognizant of surgical decisions that may minimize nerve injury. This includes division of the uterosacral and cardinal ligament as close to midline as possible and consideration of intra-fascial technique to minimize nerve injury. These steps can help elucidate the mechanism behind post-hysterectomy PFD and minimize surgical morbidity without a dramatic change to current practice.

The etiology of PFD is an evolving field with many avenues for research, and there are still fundamental questions about pelvic anatomy and innervation that remain unsolved. For many years, research on hysterectomy’s role in PFD has been stagnant, muddied by contradicting association studies and a lack of clear direction. In this paper, we have strived to address the causes of this stagnation with the hope of renewing interest in the topic. Because hysterectomy does not fit many of the well-known etiologies of PFD, its study may open doors to previously unexplored relationships in the female pelvis. We hope this encourages researchers to venture beyond existing frameworks for the etiology of PFD and believe that this work has the potential to transform our understanding of hysterectomy and the pathogenesis of pelvic floor disorders.
